# Exercise as a Promising Non-Pharmacological Intervention for ADHD

**DOI:** 10.31083/RN48058

**Published:** 2025-12-22

**Authors:** Sergio Machado, Flávia Paes, João Lucas Lima

**Affiliations:** ^1^Center for Neuroscience, Neurodiversity Institute, Nova Iguaçu, RJ 26210-270, Brazil; ^2^Panic and Respiration Laboratory (LABPR) - Institute of Psychiatry (IPUB), Federal University of Rio de Janeiro (UFRJ), Rio de Janeiro, RJ 22290-140, Brazil; ^3^Virtual Reality in Mental Health Laboratory (RVSM) - Institute of Psychiatry (IPUB), Federal University of Rio de Janeiro (UFRJ), Rio de Janeiro, RJ 22290-140, Brazil

Attention-deficit/hyperactivity disorder is one of psychiatry’s most complex neurodevelopmental challenges, marked by its characteristic triad of inattention, hyperactivity, and impulsivity that often extends well beyond childhood into adult life [1]. The psychiatric community continues to rely heavily on pharmacological approaches, with stimulant medications maintaining their first-line status, yet we must acknowledge their growing list of limitations. In our clinical experience, the variable patient response patterns, troublesome side effect profiles, and concerning lack of impact on physical health complications, collectively underscore an urgent need for complementary treatment paradigms [2, 3]. Within this evolving therapeutic landscape, we have seen physical exercise emerging as a particularly promising non-pharmacological alternative. Our research group has accumulated evidence for its beneficial impacts on both the cognitive and behavioral manifestations of attention-deficit hiperativity disorder (ADHD) [4].

The neurobiological pathways through which exercise modulates ADHD symptoms are both fascinating and complex. Our analysis of the current literature reveals that both single exercise sessions and sustained exercise regimens appear to fine-tune catecholaminergic systems, with particular emphasis on dopamine and noradrenaline pathways that form the crux of ADHD’s pathophysiology [5]. The evidence we have compiled, drawing from both preclinical models and human trials, consistently indicates that physical exertion stimulates the release of these essential neurotransmitters while simultaneously increasing serotonin availability and brain-derived neurotrophic factor concentrations, thereby fostering neuronal plasticity and strengthening cognitive functions [6, 7]. Our examination of advanced neuroimaging data further reinforces these observations, demonstrating that consistent exercise results in both structural reorganization and functional optimization within brain regions most commonly implicated in ADHD, especially the prefrontal cortex and hippocampal complex [8, 9]. Synthesizing these diverse investigative approaches, we hypothesize that exercise may effectively counter core ADHD deficits by accessing the same neural circuitry targeted by stimulant pharmacotherapy, albeit through the brain’s intrinsic regulatory systems rather than external chemical manipulation.

Our research team has been particularly intrigued by recent findings highlighting the rapid cognitive enhancements following discrete periods of exercise, especially within attention and inhibitory control parameters. In pediatric ADHD populations, we have documented how moderate-intensity aerobic activities, typically structured around 20–30 minutes of cycling or running, reliably boost performance on cognitive challenges including the Flanker and Stroop paradigms. These behavioral improvements frequently coincide with increased P300 amplitude in electrophysiological recordings, indicating more efficient allocation of attentional resources [10, 11]. Although the results in adult populations show greater variability, certain exercise modalities demonstrate particular promise. High-intensity interval training (HIIT) appears effective in reducing hyperactivity and improving mood, while yoga practice seems to enhance delayed reward processing [4, 12, 13]. Our interpretation of the acute effects suggests that they likely result from transient neurochemical changes, which makes exercise a potential cognitive enhancer in daily routines. Despite substantial evidence supporting its acute benefits, the literature investigating the long-term effects of exercise remains limited and the methodology is diverse. Studies involving children with ADHD reveal considerable variation in results, largely attributed to differences in program design and implementation [11, 12, 13]. The investigation by Choi and colleagues [14] exemplifies positive outcomes, in which a 6-week mixed-exercise program administered three times weekly alongside methylphenidate yielded significant improvements in inattention and executive functions. In contrast, Ziereis and Jansen [15] conducted a comparison between a specialized coordination training program, emphasizing balance and ball skills and a generic exercise regimen over 12 weeks. Interestingly, both approaches produced comparable improvements in verbal and spatial working memory. This finding leads us to consider whether the structured nature of regular exercise, regardless of its specific form, might represent the crucial element for cognitive enhancement.

The diversity in methodology observed in these studies offers a valuable window through which to interpret the heterogeneous results in the literature. Our analysis suggests that distinct programs can activate shared beneficial pathways, provided that the interventions maintain adequate structure and consistency in their application. This perspective forces us to reconsider previous assumptions about the specificity of training, highlighting instead the intrinsic value of regular and systematic practice. While mixed programs incorporating elements such as taekwondo or yoga demonstrate benefits for executive functions and social behavior, outcomes continue to vary substantially across various studies [14, 15]. A particularly significant research gap concerns the absence of long-term exercise studies in adults with ADHD, a notable limitation given the disorder’s chronic trajectory. Persistent methodological challenges, such as small sample sizes, inadequate control groups, and heterogeneity in exercise protocols, significantly compromise the interpretation of these findings. It is crucial that future research focus on randomized clinical trials with established methodologies, which are essential for unraveling dose-response relationships and establishing and optimizing exercise programs. The growing body of evidence on exercise in ADHD raises profound clinical questions while exposing substantial conceptual gaps. Our analyses show that moderate-intensity aerobic activities, particularly sessions lasting approximately 30 minutes, can synergistically complement conventional approaches by improving mechanisms of attention, inhibitory control, and emotional modulation, which can protect patients from adverse events associated with pharmacotherapy. These results highlight the pressing need in clinical practice to integrate structured exercise programs into individualized treatment plans, particularly for individuals with residual symptoms or drug intolerance. This approach represents not only a complementary strategy, but also involves rehabilitative programs that deserve greater recognition in our therapeutic guidelines.

It is critical that exercise recommendations account for developmental differences between various populations. Exergaming provides children with an engaging combination of physical exertion and interactive play, thereby promoting adherence while simultaneously exercising cognitive-motor functions [16]. Adults, however, may find High-Intensity Interval Training more practicable due to its time-efficient, and variable nature, that aligns well with busy schedules and helps counteract monotony, a common adherence barrier in this population [12, 13]. Nevertheless, the heterogeneity evident across existing studies necessitates a more nuanced application. While aerobic exercise appears particularly beneficial for attention, emerging evidence suggests coordinative activities like yoga or taekwondo might more directly address impulsivity and motor deficits [15, 17]. This perspective was supported by Demircioğlu *et al*. [18], who documented clear motor impairments in children with combined-type ADHD compared to typically developing peers, including deficits in bilateral coordination, balance, speed, and agility. Gait analysis further revealed a prolonged swing phase in the ADHD group, with impairments in upper limb coordination and balance directly affecting gait speed and stride length. This evidence highlights the clinical relevance of incorporating objective motor assessments into evaluation protocols, allowing for more targeted and precise interventions. From a practical perspective, this variability in results indicates that exercise prescriptions need to be customized to individual symptom profiles and personal preferences, maximizing both adherence and effectiveness.

Addressing practical barriers, including motivational challenges and limited access to adequate facilities, remains equally crucial, with community initiatives and school-based programs potentially playing a key role in maintaining consistent engagement. Several key areas require focused attention in future research. First, large-scale, rigorously designed randomized controlled trials are needed to establish optimal exercise parameters regarding the type, duration, and intensity, particularly for adults with ADHD, who are severely underrepresented in the current literature [4, 13]. Second, mechanistic studies employing advanced neuroimaging techniques should elucidate how exercise induces neuroplastic changes in brain networks implicated in ADHD [4, 19]. Third, the relationship between exercise and common comorbidities, including anxiety, depression, and obesity, deserves further examination, since exercise may offer integrated management for these overlapping conditions [12, 20]. Finally, translational initiatives are essential to bridge the research-practice gap, requiring collaborative efforts among educators, policymakers, and healthcare professionals to successfully integrate exercise into standard ADHD care [4, 21]. Addressing these priorities will enable the refinement of exercise-based interventions and the full realization of their potential as reliable and cost-effective components of ADHD management.

In conclusion, exercise continues to be a low-risk, high-reward intervention for ADHD, with demonstrated benefits across cognitive, behavioral, and brain function domains [4, 10, 11]. Despite persistent methodological limitations, cumulative evidence supports its inclusion in treatment guidelines. Through continued rigorous research and thoughtful implementation, we can better utilize exercise to improve the lives of individuals with ADHD. In the future, researchers should also explore combined intervention strategies, such as exercise paired with neuromodulation approaches such as repetitive transcranial magnetic stimulation (rTMS). A recent study by Tian and colleagues [22] found that rTMS, combined with pharmacotherapy, yielded significant improvements in children with ADHD, suggesting promising synergy between these treatment modalities. We hypothesize that exercise creates an optimal neuroplastic window for subsequent neuromodulation. There is strong evidence that a single session of moderate aerobic exercise not only elevates levels of key neurotransmitters but also induces a state of enhanced neural plasticity, characterized by increased BDNF expression and more efficient functioning of cortical networks [4]. This window of opportunity, which lasts approximately 30 to 60 minutes after exercise, can be strategically exploited: if exercise prepares the neurochemical and physiological groundwork, techniques such as TMS or transcranial direct current stimulation (tDCS) can then precisely modulate specific frontostriatal circuits. A sequential approach with exercise preceding neuromodulation might therefore optimize the brain’s state to support more efficient and enduring neuroplastic changes. Rather than applying isolated interventions, this two-stage synergistic strategy could more effectively normalize executive control circuits, offering new possibilities for integrated treatment protocols in ADHD [23].
